# Efficacy of Credelio Quattro^™^ (lotilaner, moxidectin, pyrantel, praziquantel) and Credelio^™^ (lotilaner) against longhorned tick, *Haemaphysalis longicornis*,-induced infestations on dogs

**DOI:** 10.1186/s13071-025-06965-y

**Published:** 2025-08-13

**Authors:** Scott Wiseman, Molly D. Savadelis, Riaan Maree, Mandie Momberg, Liezl Whitehead, Lisa Young

**Affiliations:** 1 Elanco Animal Health, Bartley Way, Hook, RG27 9XA UK; 2 Elanco Animal Health, Innovation Way, Greenfield, IN 46140 USA; 3 Clinvet , Waverly, NY 14892 USA; 4 Clinvet, Bloemfontein, South Africa

**Keywords:** Credelio Quattro, *Haemaphysalis longicornis*, Ixodidae, Lotilaner, Credelio, Longhorned tick

## Abstract

**Background:**

*Haemaphysalis longicornis*, the longhorned tick, is an invasive tick species that has been identified in increasing numbers and regions across the USA. This tick species is a competent vector for various pathogens to dogs, humans, and other species, with heavy infestations documented to lead to exsanguination. Therefore, determination of ectoparasiticides providing adequate treatment and control of *H. longicornis* is imperative to help reduce vector-borne disease transmission and protect against infestation in dogs.

**Methods:**

Three laboratory studies were conducted to evaluate the efficacy of Credelio Quattro and Credelio for the treatment and control of *H. longicornis.* A total of 30 dogs per study were randomized to receive either placebo, Credelio Quattro, or Credelio on Day 0 according to a complete block design on the basis of pre-treatment live attached *H. longicornis* counts, infested on Day −7. To assess efficacy against preexisting infestations, enrolled dogs were infested with 50 unfed adult *H. longicornis* on Day −2 or −1 prior to treatment. Residual efficacy post-treatment was evaluated with subsequent infestations on Days 5, 12, 19, and 30. All ticks were collected and evaluated as live or dead and free or attached 48 h after treatment or infestation.

**Results:**

Adequacy of infestation was achieved in at least two studies for every infestation time point evaluated. Both Credelio Quattro and Credelio provided 100% efficacy against *H. longicornis* from Day 2 through Day 32, with no live ticks observed on any dogs. A statistically significant number of dead ticks were recovered from both treated groups as compared with control on all assessment days. Post-treatment, treatment-related diarrhea was reported in six dogs receiving Credelio Quattro and one dog receiving Credelio on Day 0, with all dogs recovering on Day 1.

**Conclusions:**

The laboratory studies described confirm the safety and effectiveness of a single dose of Credelio Quattro and Credelio, at the minimum effective dosage of 20 mg/kg lotilaner, 0.02 mg/kg moxidectin, 5 mg/kg praziquantel, 5 mg/kg pyrantel, and 20 mg/kg lotilaner, respectively, for the treatment and control of *H. longicornis* infestations in dogs for one month.

**Graphical abstract:**

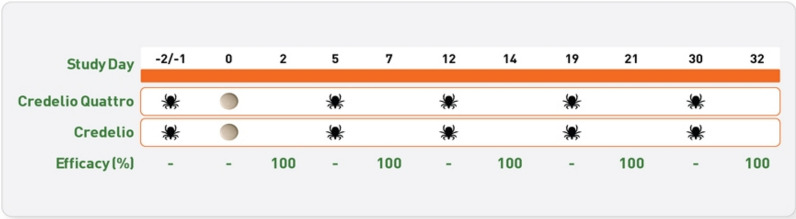

## Background

*Haemaphysalis longicornis*, the longhorned tick, is an invasive tick species in the USA. Introduction of *H. longicornis* to the USA is hypothesized to have originated from a Northeast Asian region [1]. Since the first confirmed identification of *H. longicornis* in New Jersey in 2017, this tick has subsequently been reported as far west as Arkansas, Missouri and Oklahoma and as far south as Georgia [2–5]. The US Department of Agriculture’s Animal and Plant Health Inspection Service (USDA APHIS) published as of January 2024 that *H. longicornis* has been confirmed in 20 states and has been documented on a wide range of hosts, such as cows, white-tailed deer, dogs, humans, raccoons, and birds [6].

*Haemaphysalis longicornis*, a three host Ixodid tick, flourishes in warm humid climates, enduring temperatures from 28.4 ° F to 104 ° F [7]. Seasonality of *H. longicornis* in the USA includes high activity in the summer months (June–August), similar to *H. longicornis* populations in other geographies [8]. This tick prefers to reside in long grasses and forested areas [9].

*Haemaphysalis longicornis* poses a significant threat to animal and human health [10]. Endemic in Eastern Asia, this tick has invaded Australia, New Zealand, and the USA. Several vector-borne diseases with zoonotic potential in humans are capable of being transmitted by *H. longicornis*, including thrombocytopenia syndrome virus causing hemorrhagic fever, tick-borne encephalitis virus as well as infections with *Anaplasma*, *Bartonella*, *Borrelia*, *Babesia*, *Ehrlichia*, and *Rickettsia* [7, 10–12]. While other Ixodid tick species, such as *Ixodes scapularis*, *Dermacentor variabilis*, or *Amblyomma* spp., are currently more important vectors of zoonotic disease in the USA, the longhorned tick’s ability to transmit a broad range of pathogens, including those causing hemorrhagic fever and tick-borne encephalitis, make it a serious concern [7]. This is especially true given its established role as a primary disease vector in Asia [13].

Very few tick species globally have documented parthenogenetic and bisexual populations, as observed with *H. longicornis.* Throughout Eastern Asia, both populations of *H. longicornis* are variably detected, while only parthenogenetic populations are present in Oceania, Shanghai city, and Sichuan Province in China and the USA, respectively [14–16]. The parthenogenic reproductive ability of *H. longicornis*, in addition to this tick’s ability to overwinter and survive in temperate environments and diverse host ranges, indicate population numbers and geographic ranges will continue to expand throughout the USA [16].

Control strategies to reduce tick populations and transmission of vector-borne diseases include reducing tick exposure through elimination of suitable tick habitats, wearing long shirts, pants tucked into socks, and use of acaricidal products. The recent development and introduction of the novel drug class, isoxazolines, has provided veterinarians and pet owners a safe ectoparasiticide capable of providing broad spectrum efficacy in dogs and cats. Although isoxazolines (lotilaner, afoxolaner, fluralaner, and sarolaner) are available globally in various formulations [17] and have been studied against *H. longicornis* on dogs [18–22]; published data on the invasive USA isolates are limited [23].

The objective of the studies described below was to evaluate the efficacy of a novel isoxazoline combination chewable tablet, containing lotilaner, moxidectin, praziquantel, and pyrantel (Credelio Quattro™, Elanco Animal Health, Greenfield, IN, USA) and lotilaner only (Credelio^™^, Elanco Animal Health, Greenfield, IN, USA), for the treatment and control of *H. longicornis* in dogs.

## Methods

Three studies were conducted to evaluate the efficacy of Credelio Quattro and Credelio for the treatment and control of *H. longicornis.* All studies were conducted according to VICH GL9 Good Clinical Practice and World Association for the Advancement of Veterinary Parasitology (WAAVP) guidelines for evaluating the efficacy of parasiticides for the treatment, prevention, and control of flea and tick infestations on dogs and cats [24]. The protocols were reviewed and approved by the study site’s Animal Care and Use Committee prior to initiation.

### Animals

Beagle dogs ranging from 14 months to 7 years of age, intact or neutered, and weighing ≥ 3.8 kg at the time of treatment were enrolled. Animals were confirmed healthy with no known history of serious disease prior to treatment and confirmed not to have been treated with any endectocides or ectoparasiticides prior to treatment that would impact the results of these studies. Dogs were individually housed after allocation to treatment groups with access to appropriate enrichment items at all times throughout the study. Age-appropriate, commercially available diets and ad libitum access to potable water were provided. Housing was environmentally controlled and a photoperiod of approximately 12-h light and 12-h darkness was maintained by overhead fluorescent lamps.

### Randomization and treatment

Dogs were randomized to receive either a negative control product (CP), Credelio Quattro, or Credelio according to a complete block design, utilizing preallocation *H. longicornis* total live, attached tick counts as a blocking factor. A total of 30 dogs with the highest preallocation tick counts, meeting all inclusion requirements, were assigned to individual housing and treatment groups.

Dogs were administered treatment orally on Day 0 in a fed state according to individual body weights collected on dDay −5. Single or a combination of tablets were administered to provide as close to the minimum target dosages of 20 mg/kg lotilaner, 0.02 mg/kg moxidectin, 5 mg/kg praziquantel, and 5 mg/kg pyrantel (Credelio Quattro) or 20 mg/kg lotilaner (Credelio) without underdosing. Placebo tablets and active tablets were similar in appearance to maintain masking.

### Experimental infestations and tick counts

**Fig. 1:**
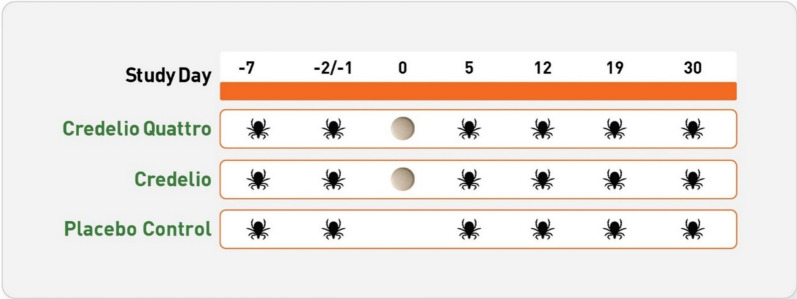
Study design of treatment administration and experimental tick infestation days

Experimental tick infestations were performed utilizing *H. longicornis* isolates obtained from New York in July 2018 (Study 1) and Virginia in October 2019 (Studies 2 and 3). To assess preallocation tick attachment rates, all 36 candidate dogs, in each study, were experimentally infested with 50 unfed adult female *H. longicornis* on Day −7. Tick counts were performed 48 h after infestation on Day −5 by combing. The total number of live, attached ticks for each dog was recorded. After enrollment and randomization, dogs were infested with 50 unfed adult female *H. longicornis* ticks on Days −2/−1, 5, 12, 19, and 30. Two studies performed infestations on Day −2 and one study performed infestations on Day −1, which were utilized to evaluate knockdown efficacy against preexisting infestations at the time of administration. All subsequent infestations evaluated residual efficacy after administration (Fig. 1). Ticks were collected by combing 48 h post-treatment on Day 2 for preexisting infestations and 48 h post-infestation on Days 7, 14, 21, and 32 after treatment. Ticks that detached and fell were collected from each dog 8-, 24-, and 48-h post-treatment or post-infestation and categorized as live or dead. The number of dead ticks at all time points in a collection period was added together to calculate the total number of dead ticks collected for evaluation of treatment.

### Safety assessments

A physical examination was performed on all dogs prior to treatment and general health observations were made at least once daily. On the day of treatment administration (Day 0) clinical observations were performed prior to treatment and at 1-, 2-, 4-, and 8-h post-treatment. General health observations were performed at least twice daily after treatment, through the end of study. All abnormal observations after the administration of treatment, whether considered to be product related or not, were documented as an adverse health event and followed until resolution. Concomitant medications administered were recorded. No dogs were exposed to any ectoparasiticide or endectocide at any time that could impact the results of these studies.

### Statistical analysis

All statistical analyses were performed utilizing SAS version 9.4 (SAS Institute, Cary, NC, US) and hypotheses were tested using two-sided tests at the 0.05 level of significance. Efficacy for Credelio Quattro and Credelio was determined post-treatment using the least squares (LS) means calculated from linear mixed models utilizing live tick counts (free and attached) as the response. Separate models were fitted to compare Credelio Quattro versus control and Credelio versus control for each assessment day and included treatment as a fixed effect and block as a random effect. The untransformed sum of dead ticks, free and attached, collected from each dog and cage were fitted using the same statistical models to assess treatment efficacy after administration of Credelio Quattro or Credelio.

## Results

Adequacy of infestation, defined by WAAVP as at least six control animals with ≥ 25% live tick attachment rate, assessed separately at each time point, was achieved in at least two of the three studies at all time points assessed (Table 1). One dog in the Credelio-treated group was excluded from Study 3 on Day 5 after a tablet was recovered underneath the dog’s caging during the 8-h post-treatment observation. LS mean of total live tick counts in the control group ranged from 11.7 to 30.8 across all time points for the three studies performed. Post-treatment, both Credelio Quattro and Credelio demonstrated 100% efficacy against *H. longicornis,* with no live attached ticks recovered from any dogs at any time points (Table 2). The difference in live tick counts was statistically significant (*p* < 0.001) when comparing both Credelio Quattro and Credelio separately with the control (Table 3).

**Table 1:** Adequacy of infestation in control treatment groups at each infestation time point

Study no	Treatment group	Number of dogs with adequate infestation (mean infestation rate_a_)				
		Day 2	Day 7	Day 14	Day 21	Day 32
1	Control	4/10_b_ (23.6%)	9/10 (47.6%)	10/10 (46.6%)	7/10 (31.6%)	6/10 (23.4%)
2	Control	9/10 (44.4%)	10/10 (61.6%)	10/10 (53.0%)	8/10 (34.2%)	5/10_b_ (23.6%)
3	Control	9/10 (47.6%)	9/10 (44.6%)	10/10 (34.8%)	10/10 (38.4%)	9/10 (34.2%)

_a_Infestation rate = (total number of ticks recovered/total number infested) × 100

_b_Adequacy of infestation criteria not met due to less than six control dogs with ≥ 25% live tick attachment rate

**Table 2:** Efficacy of Credelio Quattro and Credelio against *H. longicornis* on the basis of least squares (LS) mean total live tick counts

Study no	Treatment group	LS mean live tick counts (% efficacy)				
		Day 2	Day 7	Day 14	Day 21	Day 32
1	Control	11.8	23.8	23.3	15.8	11.7
	Credelio Quattro	0.0(100)	0.0 (100)	0.0(100)	0.0 (100)	0.0 (100)
	Credelio	0.0 (100)	0.0 (100)	0.0 (100)	0.0 (100)	0.0 (100)
2	Control	22.2	30.8	26.5	17.1	11.8
	Credelio Quattro	0.0 (100)	0.0 (100)	0.0 (100)	0.0 (100)	0.0 (100)
	Credelio	0.0 (100)	0.0 (100)	0.0 (100)	0.0 (100)	0.0 (100)
3	Control	23.8	22.3	17.4	19.2	17.1
	Credelio Quattro	0.0 (100)	0.0 (100)	0.0 (100)	0.0 (100)	0.0 (100)
	Credelio	0.0 (100)	0.0 (100)	0.0 (100)	0.0 (100)	0.0 (100)

**Table 3:** Comparison of total live *H. longicornis* tick counts on the basis of LS means for all studies combined

Day	Control versus Credelio quattro			Control versus Credelio		
	Efficacy (%)	*p*-value	Test statistic	Efficacy (%)	*p*-value	Test statistic
2	100	< 0.001	t_29_ = 11.43	100	< 0.001	t_29_ = 11.43
7	100	< 0.001	t_29_ = 14.77	100	< 0.001	t_28_ = 14.52
14	100	< 0.001	t_29_ = 17.19	100	< 0.001	t_28_ = 16.90
21	100	< 0.001	t_29_ = 11.51	100	< 0.001	t_28_ = 11.32
32	100	< 0.001	t_29_ = 10.03	100	< 0.001	t_28_ = 9.85

LS mean of total dead tick counts across all time points and studies ranged from 0.5 to 6.1 in the control group, 11.2–26.9 in the Credelio Quattro-treated group, and 10.7–29.6 in the Credelio-treated group (Table 4). The difference in total dead tick counts was statistically significant (*p* < 0.001) when comparing both Credelio Quattro and Credelio separately with the control (Table 5).

**Table 4:** LS mean of total dead *H. longicornis* tick counts recovered

Study no	Treatment group	LS mean dead tick counts				
		Day 2	Day 7	Day 14	Day 21	Day 32
1	Control	2.9	1.5	2.5	6.1	3.9
	Credelio Quattro	16.8	19.3	19.8	26.9	13.1
	Credelio	19.2	23.8	20.1	25.0	12.6
2	Control	0.9	1.5	1.1	0.5	1.2
	Credelio Quattro	25.7	24.0	16.5	11.7	11.2
	Credelio	29.6	24.7	18.3	11.4	15.4
3	Control	0.9	4.3	1.3	1.8	2.8
	Credelio Quattro	17.9	15.5	14.4	11.7	17.2
	Credelio	21.7	15.8	14.0	10.7	12.4

**Table 5:** Comparison of total dead *H. longicornis* tick counts on the basis of LS means for all studies combined

Day	Control LS mean	Control versus Credelio quattro			Control versus Credelio		
		LS mean	*p*-value	Test statistic	LS mean	*p*-value	Test statistic
2	1.6	20.1	< 0.001	t_29_ = 11.68	23.5	< 0.001	t_29_ = 12.58
7	2.4	19.6	< 0.001	t_29_ = 12.56	21.7	< 0.001	t_28_ = 12.81
14	1.6	16.9	< 0.001	t_29_ = 13.06	17.6	< 0.001	t_28_ = 14.37
21	2.8	16.8	< 0.001	t_29_ = 8.95	15.9	< 0.001	t_28_ = 7.72
32	2.6	13.8	< 0.001	t_29_ = 9.01	13.5	< 0.001	t_28_ = 7.85

Post-treatment on Day 0, diarrhea was reported in six dogs treated with Credelio Quattro (five dogs in Study 1 and one dog in Study 3) and one dog treated with Credelio (Study 1) which were likely related to treatment administration. Dogs in Study 1 were treated with butafosfan and probiotics and all study dogs recovered on Day 1. The single episode of diarrhea in a dog in Study 3 resolved without treatment. A second episode of diarrhea was reported in one dog nine days post-treatment with Credelio Quattro in Study 1 that was treated with probiotics and recovered on Day 11. No definitive cause for the diarrhea on Day 9 was determined but it was assessed as unlikely related to treatment administration.

## Discussion

Lotilaner, administered alone (Credelio) or in combination with moxidectin, praziquantel and pyrantel (Credelio Quattro) demonstrated 100% effectiveness against *H. longicornis* for one month. Isoxazolines demonstrate robust acaricidal activity but with varying speed-of-kill through the entirety of the treatment duration. The ability of ectoparasiticides to provide efficacy against ticks quickly is critical as ticks transmit various vector-borne pathogens at different rates; therefore, a product that kills ticks quickly offers the best chance of minimizing disease transmission [17, 25]. Lotilaner has a sustained speed-of-kill as demonstrated by a study evaluating the comparative speed-of-kill of Credelio, Simparica Trio^®^ (sarolaner, Zoetis, Kalamazoo, MI, USA) and NexGard^®^ (afoxolaner, Boehringer Ingelheim, Duluth, GA, USA) against *A. americanum*, a tick capable of transmitting various vector-borne diseases such as *Ehrlichia* spp, *Rickettsia* spp., and *Cytauxzoon felis* and is described as an aggressive feeder. In this study, both Credelio and NexGard achieved efficacy > 90% at 24-h post-treatment while Simparica Trio did not achieve > 90% until 48-h post-treatment [26]. At 21 days after treatment administration, Credelio demonstrated > 90% efficacy within 24-h post-infestation, while Simparica Trio and NexGard did not achieve > 90% efficacy until 48- and 72-h post-infestation, respectively [26]. In addition, 28 days after treatment administration, Credelio demonstrated > 90% efficacy at 24-h post-infestation, Simparica Trio at 72-h post-infestation and NexGard only reaching 86.3% efficacy at 72-h post-infestation [26]. Lotilaner, the active ingredient in both Credelio and Credelio Quattro, maintained a consistent speed-of-kill against *A. americanum* ticks over the entire month in this comparative study. This contrasts with Simparica Trio and NexGard, which showed declining efficacy during the month. While this study was against a different tick species than the primary efficacy studies presented here, the results suggest lotilaner’s speed-of-kill performance would be sustained against other Ixodid tick species.

Implementation of highly effective products for the treatment and control of ectoparasites is an important component of successful control strategies. The use of a single dose of Credelio Quattro or Credelio, demonstrated 100% efficacy against *H. longicornis* when evaluated either 48-h after treatment administration or 48-h after infestation post-treatment, with no live ticks attached on any treated dog at any time point. Due to the parthenogenic ability of *H. longicornis,*, even a single live female can reproduce [27]. Therefore, by providing complete efficacy in dogs, administration of Credelio Quattro or Credelio may help reduce local *H. longicornis* populations and help reduce infestation of animals in the surrounding area. Additional environmental control strategies to reduce tick populations include eliminating ground vegetation, keeping grass lengths short, and controlling wildlife access. When done in combination, effective acaricidal treatments and prompt removal of any attached ticks can help reduce potential tick infestations and vector-borne disease transmission to dogs and humans alike.

## Conclusions

These laboratory studies confirm a single dose of Credelio Quattro, a novel oral combination chewable tablet at the minimum labeled dosages of 20 mg/kg lotilaner, 0.02 mg/kg moxidectin, 5 mg/kg praziquantel and 5 mg/kg pyrantel, and Credelio at the minimum dosage of 20 mg/kg lotilaner are safe and effective for the treatment and control of *H. longicornis* infestations in dogs for one month.

## Data Availability

Data supporting the conclusions of this article are included within the article.

## Abbreviations

CP: Control product
LS: Least Squares
USA: United States of America
USDA APHIS: United States Department of Agriculture Animal and Plant Health Inspection Service
WAAVP: World Association for the Advancement of Parasitology
